# Supplementary material for: PGxO and PGxLOD: a reconciliation of pharmacogenomic knowledge of various provenances, enabling further comparison
Source: BMC Bioinformatics. 2019 Apr 18;20(Suppl 4):139. doi: 10.1186/s12859-019-2693-9 (PMC6471679; doi:10.1186/s12859-019-2693-9)
Supplement: Supplementary file 1 — PGxO reconciliation rules. This PDF file provides the definition of the five reconciliation rules and illustrates their behavior with concrete examples. (PDF 337 kb) [file 12859_2019_2693_MOESM1_ESM.pdf]

Additional file 1 – PGxO reconciliation rules

Title: PGxO and PGxLOD: a reconciliation of pharmacogenomic knowledge of various provenances, enabling further comparison

Authors: Pierre Monnin, Joël Legrand, Graziella Husson, Patrice Ringot, Andon Tchechmedjiev, Clément Jonquet, Amedeo Napoli, Adrien Coulet

This document defines the reconciliation rules used with PGxO [1] for the comparison of pharmacogenomic knowledge units of various provenances. Only the necessary definitions are recalled in the next sections. For further information, please refer to the BioPortal page of PGxO[1] as well as the documentation page of PGxO on GitHub[2].

1 Definitions and notations

The `PharmacogenomicRelationship` concept is defined in Description Logic with the following axioms:

Axiom 1. $\texttt{DComponent} \equiv \texttt{Drug} \sqcup \exists \texttt{dependsOn.Drug}$

Axiom 2. $\texttt{GFComponent} \equiv \texttt{GeneticFactor} \sqcup \exists \texttt{dependsOn.GeneticFactor}$

Axiom 3. $\texttt{PR}_1 \equiv \exists \texttt{causes.Phenotype} \sqcap \exists \texttt{isCausedBy.DComponent}$

Axiom 4. $\texttt{PR}_2 \equiv \exists \texttt{causes.Phenotype} \sqcap \exists \texttt{isCausedBy.GFComponent}$

Axiom 5. $\texttt{PR}_3 \equiv \exists \texttt{isCausedBy.DComponent} \sqcap \exists \texttt{isCausedBy.GFComponent}$

Axiom 6. $\texttt{PharmacogenomicRelationship} \sqsubseteq \texttt{PR}_1 \sqcup \texttt{PR}_2 \sqcup \texttt{PR}_3$

Intuitively, it defines a `PharmacogenomicRelationship` as a relationship between three types of components: `Drug`s, `GeneticFactor`s and `Phenotype`s. At least two of the three types of components must be present so that an individual can be an instance of the `PharmacogenomicRelationship` concept.

Considering an instance `r` of the `PharmacogenomicRelationship` concept from a Knowledge Base $\mathcal{KB}$, we denote sets of individuals or classes associated to `r` as follows.

Notation 1. *We denote D, the set of instances of* `Drug` *that cause* `r`*. D is defined as follows:*

$$D = \{\texttt{d} \mid \mathcal{KB} \models \texttt{Drug(d)} \textit{ and } \mathcal{KB} \models \texttt{causes(d,r)}\}$$

Notation 2. *We denote G, the set of instances of* `GeneticFactor` *that cause* `r`*. G is defined as follows:*

$$G = \{\texttt{g} \mid \mathcal{KB} \models \texttt{GeneticFactor(g)} \textit{ and } \mathcal{KB} \models \texttt{causes(g,r)}\}$$

[1] http://bioportal.bioontology.org/ontologies/PGXO
[2] https://github.com/practikpharma/PGxO

Notation 3. *We denote P, the set of instances of* `Phenotype` *caused by* `r`. *P is defined as follows:*

$$P = \{\mathtt{p} \mid \mathcal{KB} \models \mathtt{Phenotype(p)} \textit{ and } \mathcal{KB} \models \mathtt{causes(r,p)}\}$$

Notation 4. *We denote DC, the set of classes instantiated by all the individuals in D. DC is defined as follows:*

$$DC = \{\mathcal{C} \mid \forall\ \mathtt{d} \in D,\ \mathcal{KB} \models \mathcal{C}(\mathtt{d})\}$$

Notation 5. *We denote PC, the set of classes instantiated by all the individuals in P. PC is defined as follows:*

$$PC = \{\mathcal{C} \mid \forall\ \mathtt{p} \in P,\ \mathcal{KB} \models \mathcal{C}(\mathtt{p})\}$$

Notation 6. *We denote GHP, the set of instances of* `GeneticFactor` *associated through* `hasPart` *to individuals in G. GHP is defined as follows:*

$$GHP = \{\mathtt{g} \mid \mathcal{KB} \models \mathtt{GeneticFactor(g)} \textit{ and } \exists\ v \in G,\ \mathcal{KB} \models \mathtt{hasPart(g,v)}\}$$

Intuitively, GHP contains the genes whose variants are involved in G.

Notation 7. *We denote DOP, the set of individuals associated through* `dependsOn`$^-$ *to individuals in P. DOP is defined as follows:*

$$DOP = \{\mathtt{e} \mid \exists\ p \in P,\ \mathcal{KB} \models \mathtt{dependsOn(p,e)}\}$$

2 Rules definitions

We consider $\mathtt{r}_1$ and $\mathtt{r}_2$ two instances of the `PharmacogenomicRelationship` concept. As defined in the previous section, we also consider the sets of individuals or classes associated with $\mathtt{r}_1$ (respectively with $\mathtt{r}_2$) as D_1, G_1, P_1, DC_1, PC_1, GHP_1 and DOP_1 (respectively D_2, G_2, P_2, DC_2, PC_2, GHP_2 and DOP_2).

2.1 When $\mathtt{r}_1$ and $\mathtt{r}_2$ are equivalent

Rule 1.

$$D_1 = D_2 \text{ AND } G_1 = G_2 \text{ AND } P_1 = P_2 \Rightarrow \mathtt{owl{:}sameAs}(\mathtt{r}_1, \mathtt{r}_2)$$

2.2 When $\mathtt{r}_1$ is more specific than $\mathtt{r}_2$

Rule 2 (When the three types of components exist)**.**

$$\begin{aligned}
[D_1 \neq \emptyset \text{ AND } G_1 \neq \emptyset \text{ AND } P_1 \neq \emptyset] &\text{ AND}\\
[D_1 \subseteq D_2 \text{ OR } (DC_2 \neq \emptyset \text{ AND } DC_2 \subseteq DC_1)] &\text{ AND}\\
[G_1 \subseteq G_2] &\text{ AND}\\
[P_1 \subseteq P_2 \text{ OR } (PC_2 \neq \emptyset \text{ AND } PC_2 \subseteq PC_1)] &\qquad \Rightarrow \mathtt{skos{:}broadMatch}(\mathtt{r}_1, \mathtt{r}_2)
\end{aligned}$$

Rule 3 (When one type of components is missing).

$$\begin{aligned}
&\Big([D_2 = \emptyset \text{ AND } G_1 \neq \emptyset \text{ AND } P_1 \neq \emptyset] \text{ AND} \\
&[G_1 \subseteq G_2] \text{ AND} \\
&[P_1 \subseteq P_2 \text{ OR } (PC_2 \neq \emptyset \text{ AND } PC_2 \subseteq PC_1)] \Big) \text{ OR} \\
&\Big([D_1 \neq \emptyset \text{ AND } G_2 = \emptyset \text{ AND } P_1 \neq \emptyset] \text{ AND} \\
&[D_1 \subseteq D_2 \text{ OR } (DC_2 \neq \emptyset \text{ AND } DC_2 \subseteq DC_1)] \text{ AND} \\
&[P_1 \subseteq P_2 \text{ OR } (PC_2 \neq \emptyset \text{ AND } PC_2 \subseteq PC_1)] \Big) \text{ OR} \\
&\Big([D_1 \neq \emptyset \text{ AND } G_1 \neq \emptyset \text{ AND } P_2 = \emptyset] \text{ AND} \\
&[D_1 \subseteq D_2 \text{ OR } (DC_2 \neq \emptyset \text{ AND } DC_2 \subseteq DC_1)] \text{ AND} \\
&[G_1 \subseteq G_2] \Big) \Rightarrow \texttt{skos:broadMatch}(\texttt{r}_1, \texttt{r}_2)
\end{aligned}$$

Rule 4 (When $\texttt{r}_1$ is at the variant-level and $\texttt{r}_2$ at the gene-level).

$$\begin{aligned}
&\Big[GHP_1 \neq \emptyset \text{ AND } GHP_1 \subseteq G_2 \Big] \text{ AND} \\
&\Big[\Big([D_2 = \emptyset \text{ AND } P_1 \neq \emptyset] \text{ AND} \\
&[P_1 \subseteq P_2 \text{ OR } (PC_2 \neq \emptyset \text{ AND } PC_2 \subseteq PC_1)] \Big) \text{ OR} \\
&\Big([D_1 \neq \emptyset \text{ AND } P_2 = \emptyset] \text{ AND} \\
&[D_1 \subseteq D_2 \text{ OR } (DC_2 \neq \emptyset \text{ AND } DC_2 \subseteq DC_1)] \Big) \text{ OR} \\
&\Big([D_1 \neq \emptyset \text{ AND } P_1 \neq \emptyset] \text{ AND} \\
&[D_1 \subseteq D_2 \text{ OR } (DC_2 \neq \emptyset \text{ AND } DC_2 \subseteq DC_1)] \text{ AND} \\
&[P_1 \subseteq P_2 \text{ OR } (PC_2 \neq \emptyset \text{ AND } PC_2 \subseteq PC_1)] \Big) \Big] \Rightarrow \texttt{skos:broadMatch}(\texttt{r}_1, \texttt{r}_2)
\end{aligned}$$

2.3 When $\texttt{r}_1$ and $\texttt{r}_2$ are related

Rule 5.

$$\begin{aligned}
&DOP_1 \neq \emptyset \text{ AND} \\
&[DOP_1 = DOP_2 \text{ OR } DOP_1 = D_2 \text{ OR } DOP_1 = G_2] \text{ AND} \\
&[(G_1 \neq \emptyset \text{ AND } G_1 = G_2) \text{ OR } (D_1 \neq \emptyset \text{ AND } D_1 = D_2)] \quad \Rightarrow \texttt{skos:relatedMatch}(\texttt{r}_1, \texttt{r}_2)
\end{aligned}$$

3 Examples

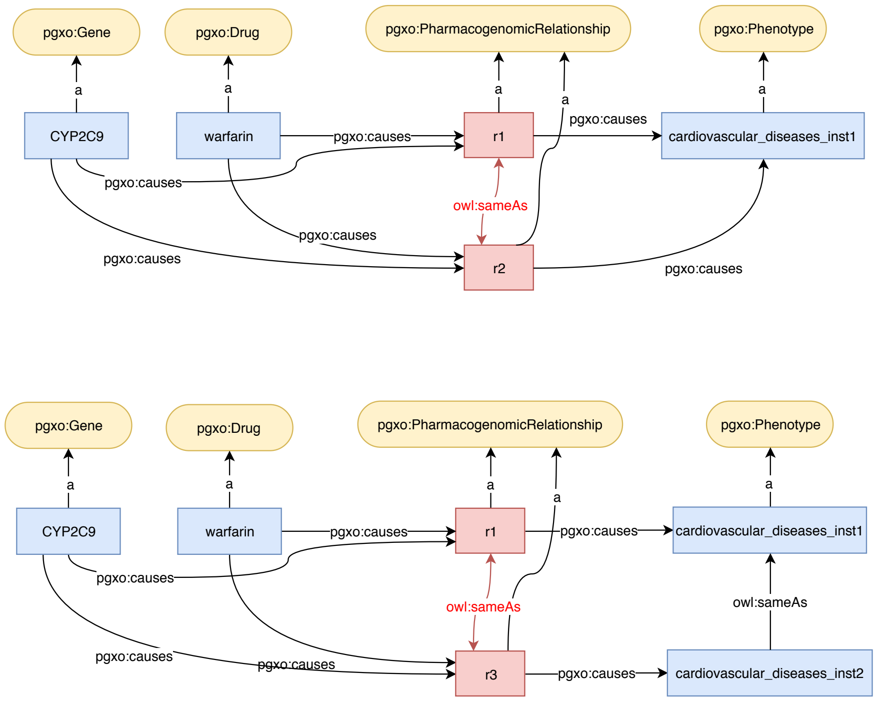

Figure 1: Examples of Rule (1) application.

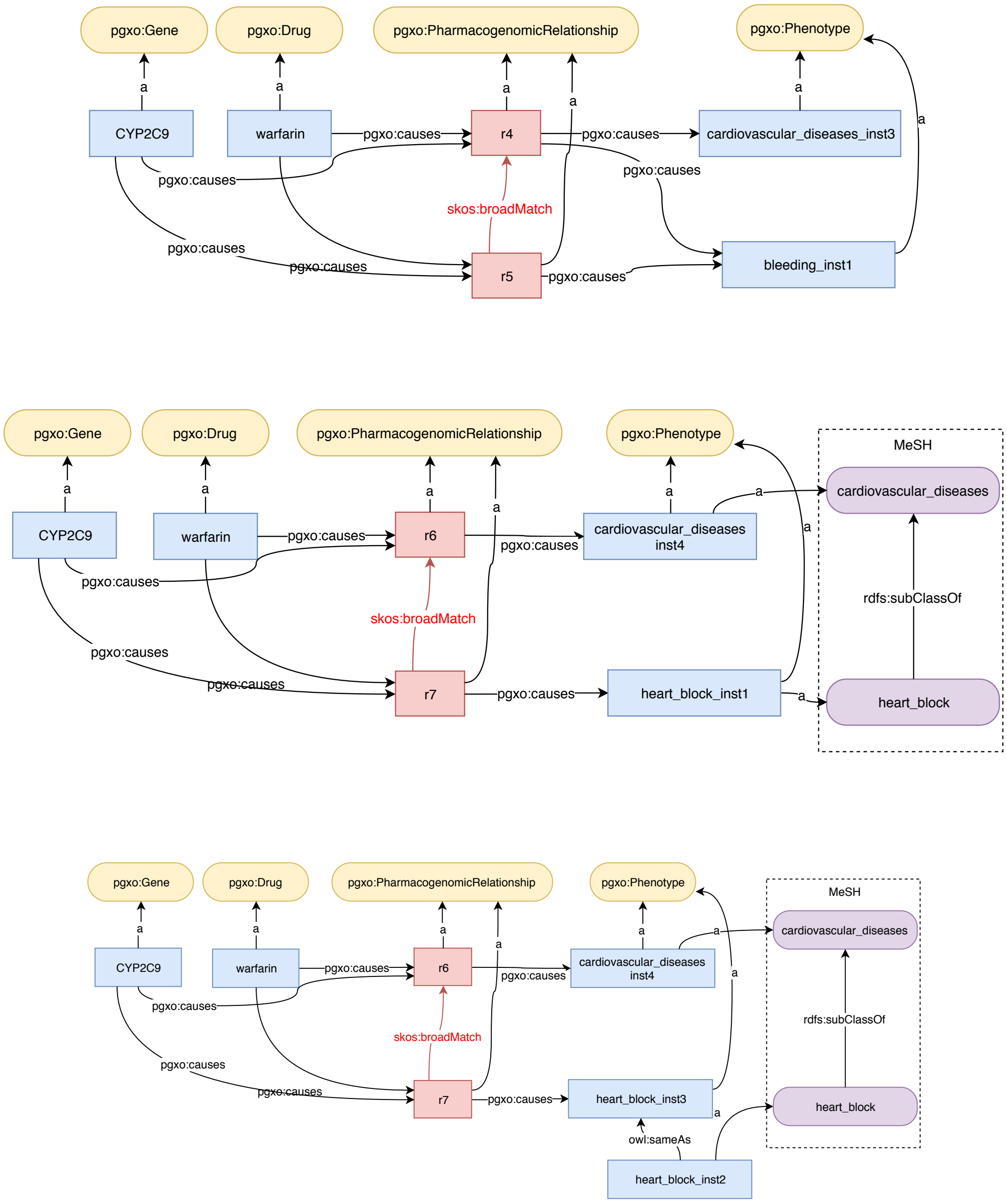

Figure 2: Examples of Rule (2) application.

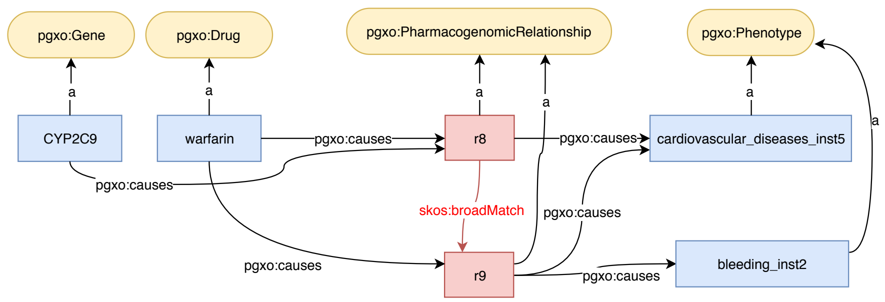

Figure 3: Example of Rule (3) application.

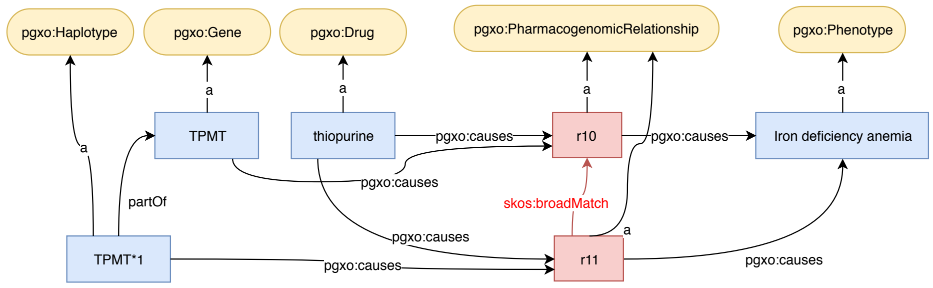

Figure 4: Example of Rule (4) application.

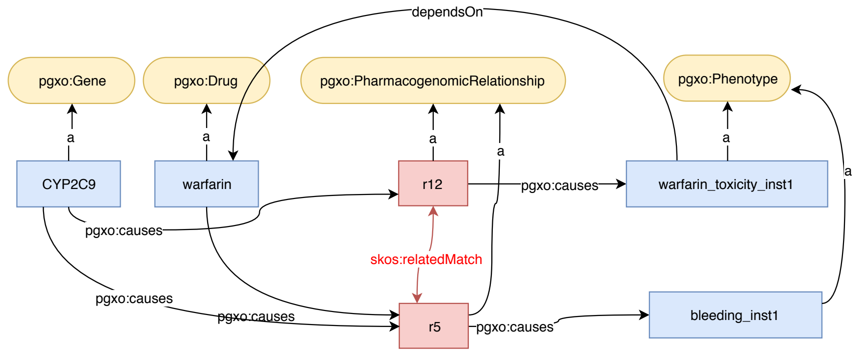

Figure 5: Example of Rule (5) application.

References

[1] Pierre Monnin, Clément Jonquet, Joël Legrand, Amedeo Napoli, and Adrien Coulet. PGxO: A very lite ontology to reconcile pharmacogenomic knowledge units. In *Methods, tools & platforms for Personalized Medicine in the Big Data Era*, 2017.
